# Commentary: Pattern Recognition Proteins: First Line of Defense Against Coronaviruses

**DOI:** 10.3389/fimmu.2022.815168

**Published:** 2022-01-26

**Authors:** Michael DePietro, Marc Salzberg

**Affiliations:** Airway Therapeutics, Marietta, GA, United States

**Keywords:** COVID-19, surfactant protein-D, viral aggregation, adult respiratory distress syndrome, bronchoalveolar lavage, SARS COV 2

## Introduction

Labarrere and Kassab recently wrote an informative paper that includes a very interesting overview of the role Surfactant Protein D (SP-D) plays as part of the innate immune system in the defense against several viral pathogens including SARS-CoV-2 (1). It has been described that the Carbohydrate Recognition Domain (CRD) of SP-D recognizes and binds to carbohydrates present in certain viruses such as the hemagglutinin of Influenza A Virus (2, 3) or the Spike (S)-Protein of SARS-CoV and SARS-Cov-2 (4, 5). Binding promotes agglutination and clearance of the virus (6). Additionally, SP-D modulates the inflammatory response induced by pathogens, SP-D interacts with specific receptors on the inflammatory cells such as the TLR4 complex, inhibiting the pro-inflammatory response induced by viruses (7–9).

Of particular interest given the SARS-CoV-2 pandemic, Labarrere and Kassab highlight several studies which have demonstrated a correlation of relatively high serum levels of SP-D with the development of severe COVID-19 pneumonia (10, 11). These findings suggest a role of Surfactant Protein D as a marker of disease severity as well as a potential therapeutic agent which Labarrere and Kassab discuss.

We would like to point out that in addition to high levels of serum levels being associated with COVID-19 severity, low levels of SP-D in the lung as measured in bronchoalveolar lavage fluid (BALF) have been reported in severe COVID-19 (5) and have been associated with an increased risk of the acute respiratory distress syndrome (ARDS) (12). These observations resulted in the filing of a New Investigational Drug (IND) application for a recombinant human surfactant Protein D (rhSP-D) formulation administered as a therapeutic agent for patients with severe COVID-19 with respiratory failure, as well as the initiation of a Phase 1B study this therapy, that is currently enrolling patients (NCT 04659122 http://www.ClinicalTrials.gov).

## Human Surfactant Protein D Is Depleted in Patients With COVID-19 Pneumonia

Arroyo et al studied 12 patients > 18 years old who had respiratory failure requiring intubation and mechanical ventilation secondary to COVID-19 (5). SARS-CoV-2 infection was confirmed with PCR testing. Patients ranged in age from 26-73, 9 men, 3 women. Bronchoscopy with bronchoalveolar lavage was done in all patients and SP-D levels were measured on the resultant aliquots of BALF using anti-hSP-D ELISA. The median SP-D level was 68.9 ng/mL, with a mean of 244.8 ng/mL. While this study did not have concurrent controls, it is notable that the levels of SP-D are substantially lower than levels which have been measured using similar methodology in healthy subjects, ranging from about 500-2000 ng/mL (13–16). Greene et al demonstrated significant lower levels of BALF SP-D in mechanically ventilated patients with ARDS who died versus survivors (12). The median BALF levels of both groups (406 ng/mL and 940 ng/mL respectively) was higher than what was observed in the COVID-19 population described above.

Arroyo et al also demonstrated that a full-length recombinant human SP-D bound the Spike protein (S-protein) of the SARS-CoV-2 virus in a calcium-dependent manner involving the carbohydrate recognition domain (5). This process also showed that protein cross bridges formed when binding the S-protein in the presence of another molecule (maltose-coated beads) suggesting the ability of SP-D to aggregate the virus and potentially facilitate viral clearance. The ability of SP-D to bind the S-protein was found in both the Wuhan variant of SARS-CoV-2 as well as several other variants tested. Recombinant human SP-D also inhibited SARS-CoV-2 viral replication in a dose dependent fashion, in human epithelial Caco-2 cells in the same study. In line with these results, Madan et al showed that a shorter recombinant fragment SP-D (rfhSP-D) was able to bind to the S-protein of SARS-CoV-2 and reduce virus infection and replication in Vero cells (17). Additional data for the fragment rfhSP-D was published by Hsieh et al also showing a dose-dependent binding of rfhSP-D to the S1-protein of SARS-CoV-2 in addition to virus entry inhibitor role for this rfhSP-D fragment (18). These two studies focused on the fragment rfhSP-D rather than the full length rhSP-D. The study by Arroyo et al is consistent with the body of evidence discussed by Labarrere and Kassab (1), documenting the role of full length rhSP-D promoting viral aggregation and clearance.

## Clinical Trial for COVID-19 Started in 2021

Based on the arguments outlined by Labarrere and Kassab and the data described above regarding the potential role of SP-D as an anti-viral therapy, our company (Airway Therapeutics) decided to pursue an IND for a full-length recombinant version of human SP-D as a treatment for patients with severe COVID-19 and respiratory failure requiring mechanical ventilation. Structurally, this full length rhSP-D is predominantly assembled as dodecamers (19) which have greater binding affinity to viruses and bacteria compared to the rfhSP-D trimeric fragment, as well as the viral aggregation and clearance activity that the fragment lacks (20, 21).

A phase 1B study was initiated earlier this year (NCT 04659122 http://www.ClinicalTrials.gov). This is a safety and dose escalation trial of 75 and 150 mg of rhSP-D administered *via* endotracheal tube (ETT) to intubated patients. The minimum dose of 75 mg is equivalent to 1 mg/kg (considering an adult weight of 70 kg) and the high dose 150 mg is equivalent to 2 mg/kg. Animal data have suggested that SP-D levels are consistent across species and increase to approximately 2 mg/kg following stress or lung injury. Experiments in sheep and mice have demonstrated efficacy of a single dose of about 0.25-2 mg/kg following a single insult such as viral infection, LPS administration, or mechanical ventilation. The doses have been selected considering the previous literature and animal studies (22, 23).

## Discussion

Beyond the above role in potential COVID-19 treatment, SP-D is known to have extensive antimicrobial and immune modulation effects. A recent review by Sørensen et al (24) describes the role of SP-D in lung function and its potential role in a broad variety of human diseases, including chronic inflammatory conditions such as chronic obstructive pulmonary disease (COPD) and asthma, illustrating the broad role that this molecule plays in the innate immune system and human biology in general. Because of the evidence for multiple important anti-inflammatory and immune-modulatory effects, rhSP-D is being evaluated for several other potential clinical uses. For example, there is significant evidence suggesting SP-D deficiency in premature neonates requiring mechanical ventilation is a risk factor to develop bronchopulmonary dysplasia (BPD), a complex disease in which lung inflammation plays an important role (22, 25). A recent review by Arroyo and Kingma outlined the potential of SP-D as a therapy for BPD and the evidence published of lung protection in appropriate pre-term animal models (23). A phase 1 study has been initiated for the treatment of respiratory failure in premature neonates to prevent BPD. (See NCT04662151 http://www.ClinicalTrials.gov).

We believe further study of this protein will shed light on important aspects of human immune function and may lead to new advances in therapy for multiple diseases and applaud Labarrere and Kassab for calling attention to this area of interest.

## Author Contributions

MD wrote the first draft of the manuscript. MS edited and revised the manuscript for important content. Both authors reread, edited, and approved the final version of the manuscript for submission.

## Conflict of Interest

The authors are executive officers for Airway Therapeutics.

## Publisher’s Note

All claims expressed in this article are solely those of the authors and do not necessarily represent those of their affiliated organizations, or those of the publisher, the editors and the reviewers. Any product that may be evaluated in this article, or claim that may be made by its manufacturer, is not guaranteed or endorsed by the publisher.
